# Using Community-Based Participatory Research Approaches to Inform Development of Culturally Appropriate Cancer Informational Materials for the Pueblo of Zuni

**DOI:** 10.1007/s13187-024-02544-4

**Published:** 2024-12-17

**Authors:** Judith Sheche, Samantha Leekity, Kassie Lalio, Cheyenne Jim, Kate Cartwright, Shiraz I. Mishra

**Affiliations:** 1 University of New Mexico Comprehensive Cancer Center, University of New Mexico Health Sciences Center, Albuquerque, NM USA; 2 Albuquerque Area Indian Health Board, Albuquerque, NM USA; 3 School of Public Administration, University of New Mexico, MSC 10 5590, Albuquerque, NM 87131 USA

**Keywords:** Breast cancer, Colorectal cancer, Cervical cancer, Cancer screening, American Indian, Health equity, Community-based participatory research, New Mexico, Cancer education

## Abstract

American Indians (AIs) experience continued disparities in incidence, mortality, and survival on cancers responsive to early screening in the USA. In New Mexico, AIs compared with other racial/ethnic populations are substantially less likely to adhere to recommended screening guidelines. Our study focuses on increasing cancer awareness using culturally, linguistically, and health literacy appropriate informational materials. We conducted 10 focus groups between June and December 2021 using non-probability purposive sampling methods in the Zuni Pueblo in rural New Mexico. We established a nine-member Tribal Advisory Panel (TAP) that included representation from tribal organizations, healthcare providers, and Zuni Pueblo leadership. We recruited TAP and other tribal members for the focus groups. The focus group questions inquired about cultural and traditional tailoring of the informational materials, specifically in terms of conveying traditional health beliefs, graphic layout, and native artwork. Focus group participants recommended designing Zuni-specific informational materials incorporating vignettes by Zuni Tribal leaders, using Zuni terms, photographs, artwork, and resources. Perspectives elicited during the focus groups informed the adaptation and development of the informational materials designed to raise awareness about cancers of the breast, colon-rectum, and cervix. These materials convey information about the cancers, their risk factors, screening options, and informational resources. The new informational materials form integral components of multi-level, multi-component interventions designed to enhance cancer screening uptake through heightened awareness about the benefits of guideline-concordant cancer screening. Designing informational materials with Zuni-specific cultural, linguistic, and tribal representation should enhance participation of Zuni Tribal members in cancer control educational interventions.

## Introduction

American Indian/Alaska Natives (AIAN) have higher incidence of colorectal and cervical cancers compared to non-Hispanic Whites (NHW) [1]. In addition, AIAN have higher rates of late-stage diagnosis for certain cancers, such as breast cancer [1, 2]. Late-stage diagnosis of cancer could result in lower 5-year survival rates. AIAN have lower 5-year survival rates than NHW for most cancers [1, 2]. In New Mexico (NM), AI individuals in the Albuquerque Area Indian Health Service (IHS) have substantially lower screening rates than the state’s NHW individuals for three screen-detectable cancers (i.e., colorectal, breast, and cervical). AI women had screening rates of 30% for breast cancer (BC) and 37% for cervical cancer (CXC) compared with 70% for BC and 78% for CXC for NHW women (personal communication [3, 4]). Colorectal cancer (CRC) screening rates are also lower for AI than NHW individuals (29% vs. 69%) [5]. These disparities in screening rates for AI men and women highlight the need to increase screening for prevention (i.e., CRC and CXC) and early detection (BC) of the three screen-detectable cancers and thus reduce disparities in incidence and mortality. We use the term Native American (NA) in lieu of AI from this point onward due to self-identification preference of the community.

Disparities in screening rates reflect a broader systemic issue. With the IHS being the main healthcare system on or near tribal lands, it presents its own unique challenges. The IHS is not a health insurance plan but relies on discretionary funds from Congress [6]. This often leaves the IHS as an underfunded healthcare system, which in turn impacts cancer screening and early detection efforts [6, 7]. This is because emergency and acute care take priority over preventive care [8]. Most IHS facilities do not have oncology programs to address cancer care [7]. This poses another potential barrier of transportation if follow-up and/or treatment is needed. Additionally, there are individual level barriers that can contribute to low screening rates. This includes whether providers recommend age-gender specific screening to their patients [9–11], as provider recommendation is an important predictor of patients receiving cancer screening. Discussing a sensitive topic like cancer may also be considered taboo within NA communities [10, 12].

The Community Preventive Services Task Force (CPSTF) recommends multi-level, multi-component interventions to increase BC, CXC, and CRC screenings by implementing evidence-based interventions that increase community demand and access or increase provider delivery of screening [13–15]. Our previous research in the Zuni Pueblo in rural western NM indicated specific preference of CPSTF interventions: the use of printed material and education [10, 12, 16]. These preferred interventions mirror the CPSTF’s recommended evidence-based interventions for increasing BC, CXC, and CRC screening. However, there are no culturally linguistically, and health literacy appropriate printed informational material that are tailored to the needs of the Zuni community.

This manuscript describes the process used to develop informational materials on BC, CRC, and CXC for the Zuni community. It builds upon our previous findings from a cross sectional community survey and focus groups that were implemented in the Zuni Pueblo [9–11, 16, 17]. We describe the process of adapting and culturally tailoring brochures, factsheets, and educational flipcharts for each of the three screen-detectable cancers using community-based participatory research (CBPR) approaches [18, 19] and data collected through focus groups.

## Participants and Methods

### Research Setting and Sample

Zuni Pueblo is a rural tribal reservation located in western NM. The reservation has approximately 7000 residents [20]. The Zuni IHS Comprehensive Health Center is located on the reservation and provides cancer screening that includes Pap/HPV tests, mammograms, and iFOBT tests. Referrals for colonoscopies are made to larger facilities with the nearest being Cibola General Hospital or Fort Defiance Indian Hospital that are within 78 miles east and 68 miles north, respectively, of the Zuni reservation. If these facilities do not have any available appointments, IHS patients are then referred to Albuquerque, NM, which is over 150 miles away. The Zuni IHS facility does not have oncology services.

Zuni Health Initiative (ZHI) staff recruited Zuni community members and members of the Tribal Advisory Panel (TAP) for focus group discussions that were held between July and December 2021. The TAP members represent different tribal programs that focus on the well-being of the community (Zuni Senior Center, IHS Womenal Advisory Panel (TAP) for focus group discussions that were held between July and December 2021. The TAP members represent different facilitiesss Center, Zuni Tribal Council, IHS Diabetes Program). We recruited community members from those who participated in a previously administered community survey and had consented to be contacted for future studies. All participants self-identified as Zuni Tribal community members.

The Zuni Pueblo Tribal Council, the Southwest Tribal Institutional Review Board (IRB) (SWT-2018–004), and the University of New Mexico Health Sciences Center IRB (HRRC # 18–264) approved the research protocol.

### Focus Group Procedures

In all, we conducted six focus groups to develop the brochures and factsheets, with 4–7 people per group (Table 1). Focus group participants consisted of both men and women in the appropriate screening age range for each cancer. We conducted two focus groups with men ages 50–75 for the development of CRC brochures and factsheets and four focus groups with women ages 21–75 for the development of BC, CXC, and CRC brochures and factsheets. We conducted these focus groups in two phases, with four focus groups in phase one and two groups in phase two. In phase one, we elicited feedback and recommendations on initial versions of the informational materials (i.e., brochures and factsheets). In phase two, we obtained feedback on the informational materials that were revised based on critique and recommendations received during the phase-one focus groups.

**Table 1:** Focus group participants

FG #	Demographic		FG mode	Cancer type	FG phase	Date	Participants
	Gender	Age					
Brochures and factsheets							
1	Women	50–75	Virtual	Colorectal	1	June 21–22, 2021	3
2	Men	50–75	In-person	Colorectal	1	June 28–29, 2021	4
3	Women	50–75	Virtual	Breast	1	July 7–8, 2021	5
4	Women	21–75	Virtual	Cervical	1	July 12–13, 2021	6
5	Women	21–75	Virtual	Colorectal, breast, cervical	2	October 4, 2021	5
6	Men	50–75	In-person	Colorectal	2	October 5, 2021	3
Total*	26						
Women	19						
Men	7						
Flipchart							
1	Women	50–75	Virtual	Colorectal	1	November 17, 2021	4
2	Women	50–75	Virtual	Breast	1	November 29, 2021	2
3	Men	50–75	In-person	Colorectal	1	November 30, 2021	3
4	Women	50–75	Virtual	Cervical	1	December 1, 2021	2
Total*	11						
Women	8						
Men	3						

^*^Includes repeat participant

We developed the flipcharts after finalization of the brochures and factsheets. For the flipchart development, we conducted one focus group or discussion per cancer type, per gender (*n* = 4 focus groups) with 2–4 people per group (Table 1). For the two groups with only two persons, we held an open discussion rather than a focus group. We conducted one focus group in-person with men ages 50–75 for discussions on the CRC flipchart, where we electronically presented the flipchart on iPads in addition to displaying a printed version. We conducted one focus group and two open discussions with women ages 50–75 on the BC, CXC, and CRC flipcharts. The majority of the focus groups and discussions were held virtually because of COVID-19 precautions in place at the time of implementation.

Before each discussion, participants were given the opportunity to review the informational material (brochures, factsheets, and flipcharts). We also shared these materials virtually during discussions. We used CBPR engagement processes to ensure that each participant provided feedback on the design and development of the informational material. We developed the focus group guides containing stem questions with follow-up probes to elicit input on the content and design of the brochures, factsheets, and flipcharts for each of the three cancers. For example, participants were first broadly asked, “what did you think?” about the material which then led to questions on comprehension of the content; layout of the content and artwork; and tailoring of the informational materials to the Zuni context. These questions included, “Is the information clear to everyone?” “How can we improve this?” “What would work best for Zuni in terms of layout?” “Would testimonials be appropriate to include for screening and cancer experiences?”.

Study team members (KK, SL, JS, CJ) conducted the focus groups, half of which were student led (KK). Three study team members are Zuni Tribal members (KK, SL, JS). Focus group sessions lasted between 1 and 1.5 h. Participants reviewed a consent letter prior to their participation and received $50 merchandize cards for their time.

### Materials

We obtained existing factsheets for the three cancers (BC, CXC, CRC) from the Albuquerque Area Southwest Tribal Epidemiology Center (AASTEC). These materials provide information on screening rates, cancer incidence, mortality, survival, and stage at diagnosis from publicly available sources such as the New Mexico Tumor Registry (NMTR), which provides NM specific data. We also obtained existing brochures on CRC and CXC developed by AASTEC. AASTEC has not developed an informational brochure on BC. The existing CRC and CXC brochures served as a template for the initial iteration of the BC brochure. We used the three existing factsheets, the two existing brochures and the newly created BC brochure to generate discussion, elicit feedback, and recommendations during the phase one focus groups.

We also developed three educational flipcharts, one for each cancer type, as part of the set of informational materials. The flipcharts were adapted from an existing CRC flipchart created by AASTEC. The flipcharts were finalized following the brochures and factsheets. Content of the flipcharts include sections on anatomy of the body, what is cancer, risk and protective factors, screening, signs and symptoms, treatment, cancer-specific incidence in the Native American population, 5-year survival rates, and local and national resources. Information, graphics, and pictures presented in the flipcharts were also obtained from publicly available resources.

## Results

### Development of Informational Materials

The phase-one focus groups provided unique insights on the cultural tailoring and linguistic and health literacy appropriateness of the informational materials. Table 2 presents the recommendations from the focus groups participants and our modifications to the brochures and factsheets. One key recommendation was to include Zuni specific pictures and artwork that reflected cultural significance. We incorporated specific photographs and artwork provided by the TAP, the A:shiwi Awan Museum and Heritage Center, and local Zuni artist Mallery Quetawki (https://quetawkiart.com/gallery/) in both the materials. Participants also recommended changes to improve comprehension of the materials. The suggested changes included removing acronyms, defining terms (i.e., in both the materials. Participants also recommend, font size and color) so as to enhance their readability. Another recommendation for the brochures was to include testimonials from Zuni leaders. We added testimonials in both English and the Zuni language in the three brochures that were provided by the TAP and community members. Lastly, focus group participants recommended we develop an additional informational material, a poster. We developed a poster based on information presented in the factsheet. Participants expressed that while the brochures and factsheets could be used for individual education, the poster could be used for community-wide education for each cancer.

**Table 2:** Changes to the informational materials

	Recommendation	Modification
Factsheets		
Cervical	• Use less medical terminology and more plain language to accommodate all reading levels • Help women determine the type of test they need based on age	• Included “What is cervical cancer” section from brochure and added anatomy images • Defined “average-risk” and hysterectomy • Added symptoms section • Created flow chart for cervical screening test
Breast	• Describe available screenings • Use less medical terminology and more plain language to accommodate all reading levels	• Included screening options with descriptions for mammogram and 3D mammography • Defined post-menopausal hormone use
Colorectal	• Keep words simple for elders to understand” • Focus on positive wording than negative (i.e., healthy weight vs. obese)	• Defined FIT/iFOBT and colonoscopy • Removed abbreviations (i.e., CRC was replaced with colorectal cancer) • Added “maintain healthy weight” for protective factors
Common changes across the 3 cancers	• Have a one-sided poster for community distribution • A smaller flipchart or brochure is easier to use in educational session • In the event of a cancer diagnosis, resources should be provided for future participants in the intervention	• Changed fact sheet format from a portrait 11 × 17 inch document to a folded booklet • Posters replace the larger 11 × 17 inch factsheets • Added local resources, including those for emotional and financial support
Brochures		
Cervical	• State FDA approval for vaccine as it reflects trust, especially since the vaccine is given to children • Place emphasis on USPSTF recommended age ranges for screening, regardless of lifestyle choices	• Added risk factors and symptoms for cervical cancer added • Added HPV vaccine language: “Three HPV vaccines approved by the FDA”; “For more information regarding the HPV vaccine contact your healthcare provider” • Defined “average-risk” • Created flow chart for cervical screening • Added quote regarding lifestyle, “It is important to continue cervical cancer screening even if you think you are too old to have a baby or are not having sex”
Breast	• “Confidential” was only in one document. There is a need for consistency in language across all documents that emphasizes “confidentiality”	• Defined postmenopausal hormone use and metastasize • Added description of mammogram and 3D mammography • Included sentence for confidentiality “Have the confidential talk with your health care provider about your personal risk of breast cancer”
Colorectal	• Have a more general look and feel so as to target both men and women • Provide updated USPSTF recommendation for age at which screening should start	• Defined FIT/iFOBT and colonoscopy • Updated age of 45 for initiation of CRC screening • Added picture of culturally significant mountain
Common changes across the 3 cancers	• Images of the specific cancer gives a better visual of what it looks like and how it grows • Testimonials are needed from Zuni members to help ease peoples’ mind • Use less medical terminology and more plain language to accommodate all reading levels	• Added testimonials in Zuni and English language that are focused on overall wellness rather than directly on specific cancers • Included anatomy images • Changed to plain language and spelled out acronyms
All informational materials (brochure, factsheet, and flipchart)		
	• Participants felt that whatever information they did not know, community members also would not know (i.e., definitions, acronyms, medical terms) • Participants self-identified as Native American than American Indian • Must show collaboration and trust by including the logos (UNMCCC and AASTEC) and the Tribal seal • Use larger and bolder font to accommodate readers of different ages and reading levels	• Added artwork and pictures that are reflective of Zuni Tribal members (landmarks, jewelry, historical photos, and community members) • Added local resources and Zuni Tribal seal • Changed wording from “American Indian” to “Native American” • Spelled out acronyms and spelled out symbols (i.e., “less than” and removed “ < ”) • Added UNM branding, changed font size and color, and added awareness month colors for each cancer

**Fig. 1:**
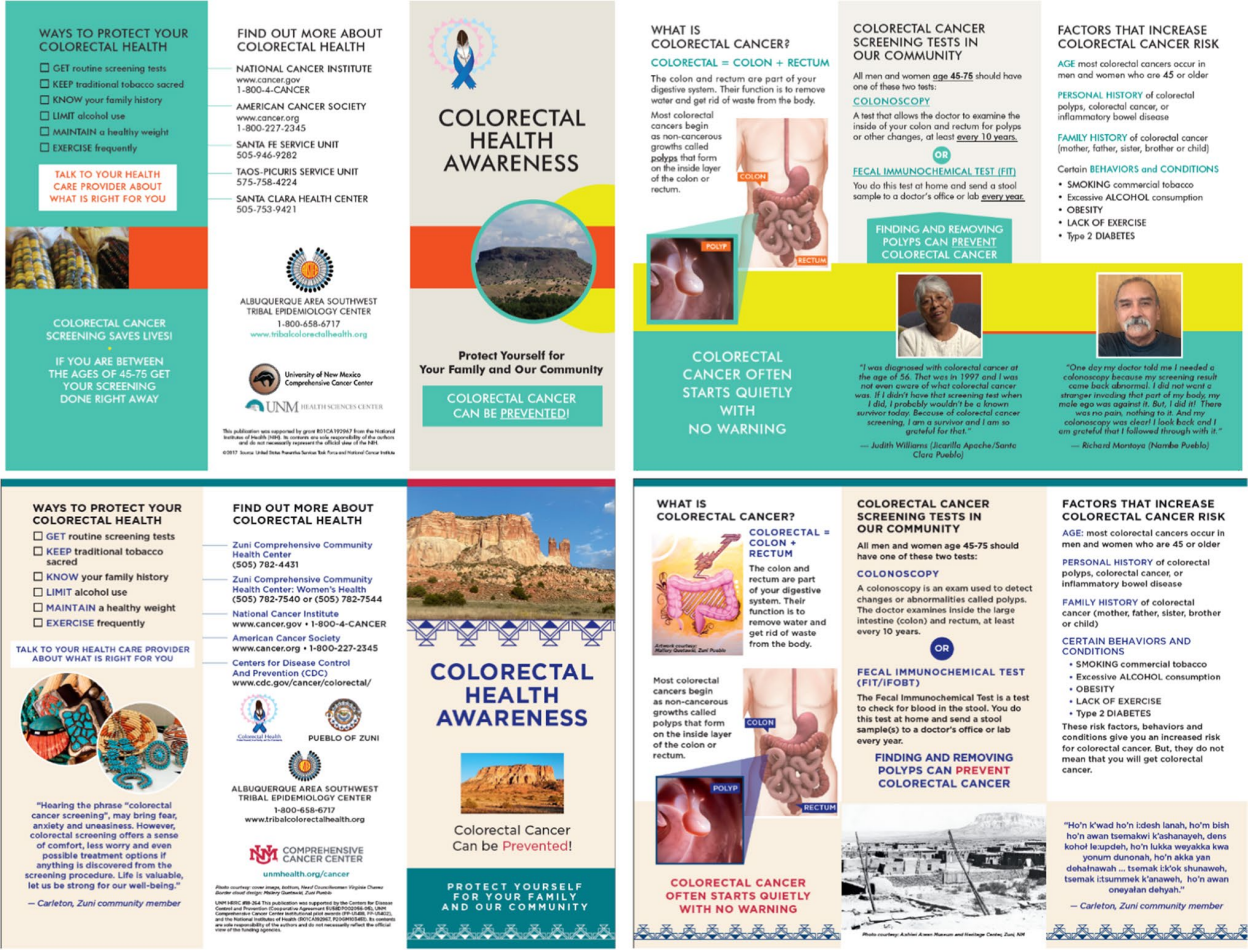
Original (top) and revised (bottom) CRC brochure

For phase-two focus groups, we presented the revised informational materials back to the participants which included their recommendations from phase one. The main recommendation during the phase-two focus groups was to align the color schemes of the brochures and factsheets to match the cancer awareness months (BC: pink, CRC: blue and CXC: teal). The UNM Comprehensive Cancer Centerolor schemes of the brochures and factsheets to match the cancer awareness months (BC: pink, CRC: blue and CXC: teal). recommendation during the phasend the Zuni la Figure 1 provides an example of the original to final revised brochure for CRC that is inclusive of all focus group feedback and University branding.

Participants of the four focus groups conducted on the initial versions of the flipcharts recommended we utilize plain-language and teach-back approaches [21] when using the flipcharts as part of our educational programs. Teach-back approaches entail asking individuals to explain back to the health educator what they have learned. The focus group participants also recommended using iPads for in-person educational sessions, as the iPads would allow participants to zoom in on graphics and pictures presented in the flipcharts. There was limited feedback on the initial versions of the flipcharts because they were developed based on the overall recommendations received during the phase-one and phase-two focus groups conducted to develop the brochures and factsheets. The flipcharts were also reviewed and approved by the UNMCCC Communication and Marketing team.

## Discussion

The purpose of this study was to develop culturally tailored, and linguistically and health literacy appropriate informational materials through active participation of the Zuni community. Our focus group participants emphasized the informational material should be tailored to best represent the importance of Zuni people and health. All informational material utilized plain-language and provided definitions, consistency in topics, and recommendations across all three cancers (i.e., including the same risk/protective factors in all materials). The language was revised to minimize creating or reinforcing fear (i.e., “scariness”) about cancer as our prior research indicated that discussing sensitive topics such as cancer is considered a cultural taboo and often related to death [10]. In all the informational materials, we have utilized positive asset-based language that has included an emphasis on wellness and health rather than being illness-focused.

With tribal communities having experienced historical mistrust, it is imperative to show existing trust in the information being provided to them. The implementation of CBPR approaches fostered this between the Zuni community, UNMCCC and AASTEC. It allowed the participants to assist in the cultural adaption of the informational material and assisted in the design of an intervention to enhance screening for the three cancers. In addition, all documents have logos for those involved to show collaboration and ongoing partnerships with the Zuni community.

Because the collaborative development of the informational materials exceeded expectations, the TAP and Tribal Council recommended the brochures be widely shared with the Zuni community. The three brochures were mailed to all active postal mailboxes in the Zuni Pueblo (*n* = 1476). Additionally, the cancer specific posters were shared with TAP members and local tribal programs for public awareness and dissemination. We also distributed all the materials to our focus group participants and the Tribal leadership (i.e., Governor and Tribal Council members). The hope is to further enhance cancer awareness in the community.

We are currently pilot testing three multi-level, multi-component interventions, one each on the three cancer types. The focus of these informational and cognitive interventions is to enhance screening uptake for the three cancers. The informational materials developed for the project forms integral components in the interventions. They are used to complement the one-on-one educational sessions conducted by study team members (SL and KH). For example, women ages 45–75 receive the three brochures and factsheets and the study team use the iPad-based flipcharts to review the three specific cancer types, its etiology, risk factors, screening options, and resources available for additional information.

As with any study, there are limitations. One data collection limitation stems from the impact of the COVID-19 pandemic on the study; due to regulations and policies implemented to mitigate harm to those in Zuni Pueblo and related health behaviors, there were some challenges to recruiting participants and individuals’ ability to participate fully in the project. An additional limitation is that the informational materials are specific to the Zuni Pueblo community experience, considering the diversity that exists in the NA population. However, through the use of CPBR and TAPs, tailoring CPSTF recommended interventions for cancer screening can be achieved using a similar process. Another limitation was the inability to publish rates on cancer incidence, mortality, survival, and screening for Zuni people that utilize the IHS in the community. Only the Tribe (Tribal government), and not the study team, can obtain local IHS utilization rates. Therefore, the factsheets and flipcharts contain data from the broader NM NA population for each cancer as reported by the Albuquerque Area IHS or NMTR.

## Conclusion

Collaborating with Zuni community members for the development of the informational material allowed for feedback from participants who had a better understanding of what would work for Zuni people. Integrating Zuni culture was essential to the development process to better convey United States Preventive Services Task Force (USPSTF) recommendations for cancer screening to the Zuni audience. These materials will facilitate education about and early detection of three screen-detectable cancers. The hope is to set forth the standard for future interventions to increase community demand and access among Native communities.

## References

[1] American Cancer Society (2022) Cancer facts and figures 2022. Accessed 16 June 2023. https://www.cancer.org/content/dam/cancer-org/research/cancer-facts-and-statistics/annual-cancer-facts-and-figures/2022/2022-cancer-facts-and-figures.pdf

[2] Kratzer TB, Jemal A, Miller KD et al (2023) Cancer statistics for American Indian and Alaska Native individuals, 2022: including increasing disparities in early onset colorectal cancer. CA A Cancer J Clin 73(2):120–146. 10.3322/caac.21757 10.3322/caac.21757 36346402

[3] New Mexico Department of Health, Indicator-based information system for public health. New Mexico’s Health Indicator Data & Statistics Cancer Screening - Cervical Cancer Screening. Published online November 27, 2020. Accessed June 16, 2023. https://ibis.doh.nm.gov/indicator/summary/CancerScrPap.html

[4] New Mexico Department of Health, Indicator-based information system for public health. New Mexico’s Health Indicator Data & Statistics Cancer Screening - Breast Cancer Screening. Published online June 28, 2021. Accessed June 16, 2023. https://ibis.doh.nm.gov/indicator/summary/CancerScrMammo.html

[5] New Mexico Department of Health, Indicator-based information system for public health. New Mexico’s Health Indicator Data & Statistics Cancer Screening - Colorectal Cancer Screening. Published online June 28, 2021. Accessed June 16, 2023. https://ibis.doh.nm.gov/indicator/summary/CancerScrColoRec.html

[6] Warne D, Frizzell LB (2014) American Indian Health Policy: historical trends and contemporary issues. Am J Public Health 104(Suppl 3):S263–S267. 10.2105/AJPH.2013.301682 24754649 10.2105/AJPH.2013.301682 PMC4035886

[7] Warne D, Kaur J, Perdue D (2012) American Indian/Alaska Native Cancer Policy: systemic approaches to reducing cancer disparities. J Canc Educ 27(S1):18–23. 10.1007/s13187-012-0315-6 10.1007/s13187-012-0315-6 22311689

[8] Indian Health Service. Purchased/referred care (PRC), eligibility, requirements: priorities of care. Accessed September 14, 2023. https://www.ihs.gov/prc/eligibility/requirements-priorities-of-care/

[9] Cartwright K, Leekity S, Sheche J et al (2023) Health Literacy, Health numeracy, and cancer screening patterns in the Zuni Pueblo: insights from and limitations of “Standard” questions. J Canc Educ 38(3):1023–1033. 10.1007/s13187-022-02227-y 10.1007/s13187-022-02227-y PMC9638364 36334245

[10] Mishra SI, Adsul P, Leekity S et al (2023) A culturally informed model to enhance breast, cervical, and colorectal cancer screenings: perspectives of American Indian adults and healthcare providers in rural New Mexico. Cancer Causes Control 34(10): 855–871. 10.1007/s10552-023-01721-y 10.1007/s10552-023-01721-y PMC10460346 37277513

[11] Cartwright K, Kosich M, Gonya M et al (2023) Cervical cancer knowledge and screening patterns in Zuni Pueblo women in the Southwest United States. J Canc Educ 38(5): 1531–1538. 10.1007/s13187-023-02295-8 10.1007/s13187-023-02295-8 PMC10097513 37046142

[12] Safi S, Ghahate D, Bobelu J et al (2022) Assessing knowledge and perceptions about cancer among American Indians of the Zuni Pueblo. NM J Canc Educ 37(6):1752–1759. 10.1007/s13187-021-02023-0 10.1007/s13187-021-02023-0 PMC8788106 33963443

[13] Community Preventive Services Task Force. Cancer screening: multicomponent interventions - cervical cancer. Published online August 2016. Accessed April 28, 2023. https://www.thecommunityguide.org/findings/cancer-screening-multicomponent-interventions-cervical-cancer.html

[14] Community Preventive Services Task Force. Cancer screening: multicomponent interventions - colorectal cancer. Published online August 2016. Accessed April 28, 2023. https://www.thecommunityguide.org/findings/cancer-screening-multicomponent-interventions-colorectal-cancer.html

[15] Community Preventive Services Task Force. Cancer screening: multicomponent interventions - breast cancer. Published online August 2016. Accessed April 28, 2023. https://www.thecommunityguide.org/findings/cancer-screening-multicomponent-interventions-breast-cancer.html

[16] Edwardson N, Kosich M, Shane Pankratz V et al (2023b) Preferences for CPSTF-recommended intervention approaches for increasing cancer screening among screen-eligible adults in Zuni Pueblo, USA. Prev Med Rep 36:102453. 10.1016/j.pmedr.2023.102453 37840594 10.1016/j.pmedr.2023.102453 PMC10568296

[17] Edwardson N, Cartwright K, Sheche J et al (2023a) Colorectal cancer screening among adults in Zuni Pueblo: factors associated with FOBT and colonoscopy utilization. J Community Health 48(4):565–575. 10.1007/s10900-023-01196-7 10.1007/s10900-023-01196-7 PMC9906599 36752868

[18] Burhansstipanov, L, Braun, KL., eds. Chapter 4: using community-based participatory research to address indigenous health. In: *Indigenous Public Health Improvement through Community-Engaged Interventions*. University Press of Kentucky; 2022:80–103

[19] Israel BA, Eng E, Schulz AJ, Parker EA. *Methods for Community-Based Participatory Research for Health*. John Wiley & Sons, Incorporated; 2012. Accessed October 11, 2023. http://ebookcentral.proquest.com/lib/unm/detail.action?docID=918182

[20] U.S. Census Bureau. American Community Survey 2021: ACS 5-year estimates subject tables for Zuni Pueblo CDP. Published 2022. Accessed October 9, 2023. https://data.census.gov/table?q=Zuni+Pueblo+CDP,+New+Mexico&tid=ACSST5Y2021.S0101

[21] Anderson KM, Leister S, De Rego R (2020) The 5Ts for teach back: an operational definition for teach-back training. HLRP: Health Literacy Research and Practice 4(2): e94–e103. 10.3928/24748307-20200318-01 10.3928/24748307-20200318-01 PMC7156258 32293689

